# Physiotherapy for sleep disturbance in chronic low back pain: a feasibility randomised controlled trial

**DOI:** 10.1186/1471-2474-11-70

**Published:** 2010-04-16

**Authors:** Deirdre A Hurley, Jennifer Eadie, Grainne O'Donoghue, Clare Kelly, Chris Lonsdale, Suzanne Guerin, Mark A Tully, Willem van Mechelen, Suzanne M McDonough, Colin AG Boreham, Conor Heneghan, Leslie Daly

**Affiliations:** 1 UCD School of Public Health, Physiotherapy & Population Science, University College Dublin, Dublin 4, Ireland; 2 UCD Institute for Sport and Health, University College Dublin, Dublin 4, Ireland; 3 Physiotherapy Department, Beaumont Hospital, Beaumont Road, Dublin 9, Ireland; 4 UCD School of Psychology, University College Dublin, Dublin 4, Ireland; 5 UKCRC Centre of Excellence for Public Health (Northern Ireland), Queens University Belfast, Mulhouse, Royal Victoria Hospital, Grosvenor Road, Belfast BT12 6BJ, Northern Ireland; 6 EMGO+ Institute for Health and Care Research, Department of Public Health and Occupational Health, VU University Medical Center, Amsterdam, The Netherlands; 7 Health and Rehabilitation Sciences Research Institute, University of Ulster, Jordanstown Campus, Antrim, BT37 0QB, Northern Ireland; 8 UCD School of Electrical, Electronic and Mechanical Engineering, University College Dublin, Dublin 4, Ireland

## Abstract

**Background:**

Sleep disturbance is becoming increasingly recognised as a clinically important symptom in people with chronic low back pain (CLBP, low back pain >12 weeks), associated with physical inactivity and depression. Current research and international clinical guidelines recommend people with CLBP assume a physically active role in their recovery to prevent chronicity, but the high prevalence of sleep disturbance in this population may be unknowingly limiting their ability to participate in exercise-based rehabilitation programmes and contributing to poor outcomes. There is currently no knowledge concerning the effectiveness of physiotherapy on sleep disturbance in people with chronic low back pain and no evidence of the feasibility of conducting randomized controlled trials that comprehensively evaluate sleep as an outcome measure in this population.

**Methods/Design:**

This study will evaluate the feasibility of a randomised controlled trial (RCT), exploring the effects of three forms of physiotherapy (supervised general exercise programme, individualized walking programme and usual physiotherapy, which will serve as the control group) on sleep quality in people with chronic low back pain. A presenting sample of 60 consenting patients will be recruited in the physiotherapy department of Beaumont Hospital, Dublin, Ireland, and randomly allocated to one of the three groups in a concealed manner. The main outcomes will be sleep quality (self-report and objective measurement), and self-reported functional disability, pain, quality of life, fear avoidance, anxiety and depression, physical activity, and patient satisfaction. Outcome will be evaluated at baseline, 3 months and 6 months. Qualitative telephone interviews will be embedded in the research design to obtain feedback from a sample of participants' about their experiences of sleep monitoring, trial participation and interventions, and to inform the design of a fully powered future RCT. Planned analysis will explore trends in the data, effect sizes and clinically important effects (quantitative data), and thematic analysis (qualitative data).

**Discussion:**

This study will evaluate the feasibility of a randomised controlled trial exploring the effects of three forms of physiotherapy (supervised general exercise programme, individualized walking programme and usual physiotherapy, which will serve as the control group) on sleep quality in people with chronic low back pain.

**Trial Registration:**

Current controlled trial ISRCTN54009836

## Background

Sleep disturbance is becoming increasingly recognised in the literature as a clinically important symptom in people with chronic low back pain (CLBP, low back pain >12 weeks) [1-5]. A large prospective study found there was a highly significant relationship between pain and sleep (P < 0.0005), with a 55% increase in the proportion of participants reporting restless/light sleep after chronic low back pain onset [2]. Sleep disturbance has been found to have a negative effect on mood, pain severity experience and general quality of life [6,7]. Depression in particular is strongly associated with disturbed sleep patterns [8,9] and has been previously identified in sleep studies involving patients with chronic LBP [10-14]. The pain literature has shown that lack of sleep or poor quality sleep lowers the pain threshold and the mental capacity to manage pain [15,16], and it has been hypothesized that better daytime pain control may lead to improved sleep quality [17]. The current literature has identified that for certain patients' chronic pain, physical inactivity, sleep disorders and depression seem to be interdependent, and whether cause or consequence, LBP-related sleep problems should be addressed as an integral part of the management plan of each patient [4].

Current research and international clinical guidelines recommend people with CLBP to assume a physically active role in their recovery to prevent chronicity [18-23], but the high prevalence of sleep disturbance in this population may be unknowingly limiting their ability to participate in exercise-based rehabilitation programmes and contributing to poor outcomes. Internationally, physiotherapy services are frequently utilized by people with LBP, predominantly for the evidence-based approaches of advice, spinal manipulative therapy and exercise therapy [23-28]. However, there is currently no knowledge of the effectiveness of any physiotherapy approaches for sleep disturbance in people with chronic low back pain.

While both subjective and objective measures of sleep patterns have been widely used in sleep research in people with various sleep disorders (e.g. obstructive sleep apnoea and insomnia) for over two decades [29,30], they have not been previously utilized as outcome measures in LBP clinical trials. Several valid and reliable subjective sleep questionnaires are available (e.g. Pittsburgh Sleep Quality Index [31], Insomnia Severity Index [32]), and the most widely used objective sleep outcome measure for research in non-laboratory settings is actigraphy [30]. Recently, other novel non-contact measurement devices have been developed to measure sleep quality in the home environment [33,34].

This project will evaluate the feasibility of undertaking a randomised controlled trial (RCT) that will explore the effects on subjective and objective measures of sleep quality and low back pain outcomes, of three forms of physiotherapy management of low back pain, i.e. a supervised general exercise class, usual physiotherapy, and a novel walking programme. The supervised exercise programme is based on the "Back to Fitness" class, whose effectiveness has been supported in several RCTs, reporting significant improvements in pain and disability compared to 'routine' physiotherapy [35] and GP management [36], with comparable effects to spinal manipulative therapy [37]. The recent UK National Institute for Clinical Excellence guideline for persistent LBP advocates such a structured exercise programme as one of the cost effective management options for this condition [23]. Recent literature has identified the need for research of brief/minimal contact self-activation interventions, such as walking programmes that encourage participation in physical activity for CLBP [22,38]. Details of the walking programme utilized in this feasibility trial have been reported in detail elsewhere [39].

The objectives are

To determine the most efficient and effective design for a main RCT by:

• Piloting the methodological procedures

• Determining the recruitment rate and actual numbers recruited

• Determining attrition rates during the intervention and follow-up periods

• Completing a qualitative exploration of participants experience of the trial procedures, interventions, and outcomes

• Evaluating the feasibility of using equipment to measure sleep disturbance within a clinical trial setting

• Determining the prevalence of sleep disturbance in the sample

• Comparing changes in sleep quality, pain, function, quality of life, fear avoidance, physical activity and patient satisfaction between groups between baseline and follow-up

• Exploring the relationship between outcome changes and sleep quality

• Conducting a power calculation to determine the numbers needed for a future large-scale, multi-centre, randomised controlled trial

• Refining the protocol for a future large-scale, multi-centre, randomised controlled trial

## Methods/Design

**Figure 1:**
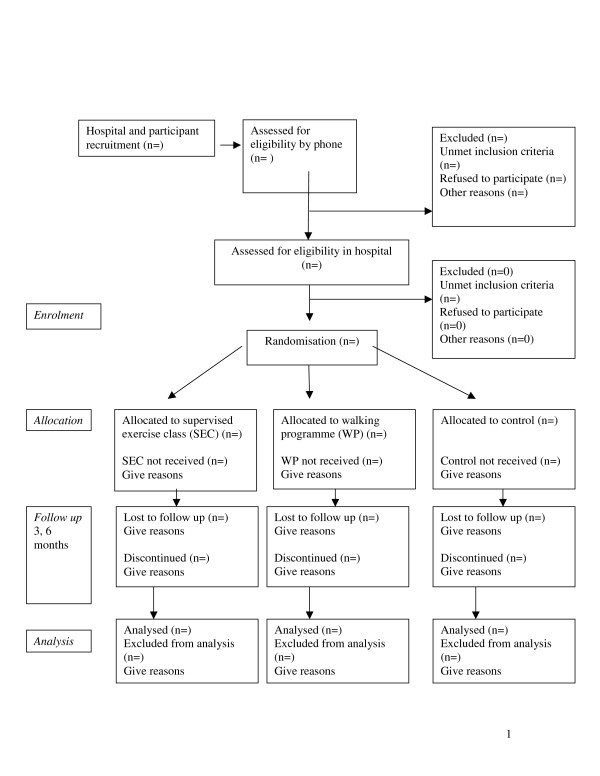
**Participant flow through the RCT (based on CONSORT statement)**.

The Research Ethics Committee of Beaumont Hospital has granted approval for this study: The trial will be reported according to the recommendations of the Consort statement [40] and the template for describing the flow of participants through the study is represented in Figure 1. The quantitative study will explore the effects of three forms of physiotherapy (i.e. a supervised general exercise programme, usual physiotherapy and a walking programme) on sleep for patients with chronic low back pain. The qualitative study will explore participants' experience of the study and the interventions.

### Quantitative study

#### Design

The study will be a prospective feasibility randomized controlled trial (RCT) with three arms (i) a supervised exercise class of 8 weeks duration (ii) a walking programme of 8 weeks duration and (iii) usual physiotherapy. Outcomes will be assessed at baseline and 3 [face-to-face] and 6 month [pre-paid postage] follow-up.

#### Controlling bias

The RCT design includes key methodological features that have been recognized as important in minimizing bias in clinical trials: true randomization, concealed allocation, specification of eligibility criteria, and intention-to-treat analysis [41].

#### Setting

A sample of patients will be recruited in Beaumont Hospital physiotherapy department that provides physiotherapy treatment for people with CLBP in the Northside of Dublin City.

#### Protocol protection

The following mechanisms will be used to ensure the trial protocol is applied consistently: protocol manuals will be developed and all involved researchers and clinicians will be trained to ensure that participant screening, assessment, random allocation and treatment procedures are conducted according to the protocol; a random sample of 10% of treatment records in each group will be audited by a researcher not involved in the day to day running of the trial to check that treatment is administered as per the protocol; if any notable anomalies are found all treatment records will be checked. The treatment record forms will be completed by clinicians on every patient recruited to the study.

### Study population and recruitment

#### Clinics and clinicians

In preparation for the ethical approval and funding applications for the trial a meeting was held with the relevant physiotherapy department manager to outline the project and distribute the outline protocol. Information meetings were held with the manager and treating physiotherapists to discuss the study background, aims and methodologies and to address their queries. Reception staff were briefed on the study recruitment process, and patient appointment systems, paperwork and filing arrangements for the trial were explored.

Prior to commencement of the trial, all participating physiotherapists involved in the active interventions (i.e. supervised exercise class, walking programme) will attend training days in the School of Public Health, Physiotherapy and Population Science, University College Dublin to be delivered by various members of the research team. The main focus of the training days will be to ensure that the interventions are standardized and a cognitive behavioural therapy (CBT) approach will be emphasized. In advance of the training days, a detailed trial manual will be distributed to the therapists and their role in the study will be highlighted.

#### Patients

All eligible participants with chronic or recurrent LBP referred to the Beaumont Hospital physiotherapy department by general practitioners or hospital consultants will be invited to participate in the study by the Trial Co-ordinator in order of referral until the target sample (n = 60) is achieved (Table 1). They will receive the initial contact by letter containing the patient information leaflet outlining the trial procedures and an invitation to attend for baseline assessment. Suitable, interested participants, once screened by telephone, will attend the physiotherapy department, where detailed verbal explanations of the study protocol will be provided and, written informed consent will be sought by the Trial Co-ordinator. The Trial Co-ordinator will record the number of participants who were invited and declined, those considered ineligible and the reasons.

**Table 1:** Eligibility Criteria

Inclusion criteria	Exclusion criteria
Patients with chronic (≥3 months) or recurrent (≥3 episodes in previous 12 months) LBP of mechanical origin with/without radiation to the lower limb	Clinically diagnosed primary sleep disorder e.g sleep apnoea, primary insomnia
Males/females between 18-70 years	Currently or having received treatment for CLBP within previous 3 months
No spinal surgery within the previous 12 months	Patients scoring <10 indicating minimum disability on the Oswestry Disability Index
Patients scoring ≥10 indicating moderate disability on the Oswestry Disability Index (ODI).	Red flags indicating serious spinal pathology, e.g. cancer, cauda equina lesion
Patients deemed suitable by their GP/hospital consultant to carry out an exercise programme	Radicular pain indicative of nerve root compression
Patients willing to attend for an 8-week treatment programme of exercise classes	Patients diagnosed with severe spinal stenosis, spondylolisthesis, fibromyalgia
Fluency in English (verbal and written)	History of systemic/inflammatory disease, e.g. rheumatoid arthritis
Access to a telephone (for follow-up support)	Patients with any confounding conditions such as a neurological disorder or currently receiving treatment for cancer
	Patients with acute (<6 weeks) or subacute LBP (6-12 weeks), provided that they have experienced <3 LBP episodes during previous 12 months
	Unstable angina/uncontrolled cardiac dysrhythmias/severe aortic stenosis/acute systemic infection accompanied by fever
	Medico-legal issues
	Pregnancy

### Eligibility assessment

#### Clinicians

Chartered Physiotherapists who are eligible for membership of the Irish Society of Chartered Physiotherapists and are employed in Beaumont Hospital are eligible to participate.

#### Patients

At the baseline interview the Trial Co-ordinator will use a screening checklist to verify eligibility (Table 1). Written signed consent will be obtained on all participants. The Physical Activity Readiness Questionnaire (PAR-Q)[42] will be completed to determine whether medical clearance is necessary before trial participation.

### Randomisation

Consenting participants will be randomly allocated in accordance with recognised procedures, by a computer-generated random allocation sequence that will be prepared centrally by the trial statistician (LD). This sequence will be used to randomly allocate each consenting, numbered participant to one of three study groups using sealed opaque envelopes: supervised exercise class, walking programme or usual physiotherapy. Prior to randomisation each participant's group allocation preference will be sought and recorded by the Trial Co-ordinator in order to investigate whether treatment preference has any influence on outcomes.

Following the baseline assessment an appointment for the relevant intervention will be made by the Trial Co-ordinator. Each participant will receive a copy of 'The Back Book'[43] and be advised to read it before the first physiotherapy appointment.

### Blinding

As this is a feasibility study blinding of the outcome assessor will not be possible but for a future main trial the outcome assessor and trial statistician would be blinded to group allocation until completion of data analysis. The Trial Co-ordinator will administer all outcome measures for face to face and postal follow-up. Due to the nature of the interventions under investigation it will not be possible to blind patients or clinicians to their group allocation in this feasibility study or a future main trial.

### Sample Size

No power-based sample size calculation has been carried out as this study aims to establish the feasibility of conducting a future multi-centre main trial of the same interventions. Recruitment will be on a consecutive basis, and will enable the researchers to estimate expected recruitment rates for the main RCT. Based on feasibility, the researchers aim to recruit 60 participants over a 6 month period at a rate of 10 per month. Based on the results of previous trials investigating physiotherapy for LBP [44], a 30% drop-out rate is anticipated between the beginning of treatment and the follow-up, so it is anticipated that n = 42 participants will complete the trial.

### Outcome measures

A combination of recommended self-report valid and reliable outcome questionnaires [45] that measure sleep quality, functional disability, pain, quality of life, physical activity, psychosocial and work loss variables, as well as objective measures of sleep quality will be completed at baseline, 3 month (face to face) and 6 month (postal) follow-up. An evaluation questionnaire regarding the utility of the objective sleep measures will be completed at baseline and the satisfaction questionnaire will only be completed at the 3 month follow-up point.

#### Baseline Assessment

At baseline, sociodemographic data (i.e. age, gender, education level, social status, occupation and work status, past medical history, LBP history), and any previous treatment for LBP will be recorded, while blood pressure, body mass index (BMI, kg/m^2^), and cardiorespiratory fitness using the shuttle walk test [46], will be measured by the Trial Co-ordinator. In addition, participants will be questioned about sleep specific factors which have been reported in the literature to influence sleep quality i.e. sleeping place (e.g. bed, chair), environmental noise (e.g. trains, airport, motorway), mattress firmness and bed-time, bed sharing, smoking, alcohol or caffeine consumption, painkillers/sleeping tablet use [8,47-53]. Each participant will also be asked to indicate their view on the primary reason for their sleep disturbance.

*Sleep quality *will be measured using three validated self-report measures (the Pittsburgh Sleep Quality Index [31] the Insomnia Severity Index [32] and the Pittsburgh Sleep Diary [54] and a validated objective measure, the Actiwatch [30].

#### Pittsburgh Sleep Quality Index (PSQI)

This is a widely used retrospective questionnaire which assesses sleep quality and disturbance over the last month [31,55-57]. Nineteen individual items generate seven "component scores" (e.g. participantive sleep quality, sleep latency, sleep duration, sleep efficiency, sleep disorders, use of hypnotics, and poor daytime functioning). Summation of these scores ('0' no difficulty-'3' severe difficulty) yields a global score (range 0-21) that indicates a participants' participantive sleep quality (score of >5 points indicative of sleep disturbance). The PSQI has a sensitivity of 89.6-98.7% and a specificity of 84.4-86.5% for identifying cases with a sleep disorder, when the cut-off score of >5 points is used. The PSQI will be the main outcome measure of sleep disturbance.

#### Insomnia Severity Index (ISI)

This questionnaire has been designed with reference to the Diagnostic and Statistical Manual for Mental Disorders (DSM-IV) criteria for insomnia and is a reliable and valid measure containing seven items to quantify perceived insomnia severity (initial, middle, terminal), dissatisfaction with current sleep pattern, interference with daily functioning, noticeability of sleep impairment attributable to the sleep problem, and degree of distress or concern raised by the sleep problem [32,58]. The statements are scored on 4-point Likert-scales ('0' not at all - '4' extremely) generating a total score (range 0-28), indicating clinical insomnia severity (0-7 points no clinically significant insomnia; 8-14 points subthreshold insomnia; 15-21 points clinical insomnia (moderate); 22-28 points clinical insomnia (severe)).

#### Pittsburgh Sleep Diary (PSD)

This is a widely used instrument with eight components, five of which are completed at bedtime and relate to the events of the day preceding the sleep (e.g. alcohol, caffeine, medication consumption, exercise, 'lights out'), and three which are completed on wakening that relate to the sleep period just completed (final wakening time, number of awakenings, sleep quality) [54]. Participants will be instructed to report every item every day for 7-nights at baseline (starting on day of recruitment) and at 3 and 6 month follow-up.

#### Actiwatch (Model AW4, CamNTech, Cambridge, UK)

This is a small wrist-mounted device (37 × 29 × 10 mm; 16 grams), worn on the non-dominant wrist, which detects and logs movement intensity and duration by means of a small piezo-electric accelerometer. Using sleep analysis software it is capable of evaluating sleepwake patterns and common sleep quality variables: i.e. sleep onset latency, sleep efficiency and sleep fragmentation. It has been extensively validated against polysomnography, the 'gold standard' for sleep studies in healthy participants [59-62] and those with sleep disorders [63-65]. The Actiwatches will be set to average activity count data during recording at 10-sec epochs. Each participant will be requested to wear the Actiwatch on two separate occasions each lasting 7 nights as recommended by the American Academy of Sleep Medicine [66] at (i) baseline (starting the first night of recruitment), and (ii) 3 month follow-up either all day or from at least 30 min before they go to bed until at least 30 min after they wake up. After each 7-night period of sleep recording, participants will return the device to Beaumont Hospital in person or by courier, where data will be downloaded for analysis using the USB Actiwatch-reader and the Actiwatch Sleep v7.27 analysis software (CamNTech, Cambridge, UK). Before analysis, actigraphic data will be automatically converted to 30-sec epochs by the software and all sleep episodes will be visually inspected (scale: 1000) to screen for malfunctioning of the devices and non-wear time.

#### Oswestry Disability Questionnaire

This is a valid and reliable measure of functional disability due to low back pain [67]. It consists of ten items, which measures participants levels of functioning/disability in various activities of daily living. Each item contains six statements (0-5 points), of which one has to be chosen. The total score is converted into a percentage score (Oswestry Disability Index, ODI) [68] with 0-20% indicating minimal disability, 21-40% moderate disability, 41-60% severe disability, 61-80% crippled and 81-100% total incapacitation.

The ODI will be the main outcome measure of LBP-related disability.

#### Numerical Pain Rating Scale

This is a widely used valid and reliable measure of pain, whereby participants will be requested to choose a number from 0 (no pain) to 10 (worst possible pain) that best describes each of the following symptoms: current and average pain for both back and leg pain [69].

#### Short-Form 36 Version 2 questionnaire (SF-36v2)

The SF-36v2 (SF-36 Medical Outcomes Trust, Waltham, MA, USA) [70], is a 36 item valid and reliable measure of health-related quality of life. It yields an eight scale profile of functional health and well-being score of which two standardised summary scores (i.e. Physical Component Score [PCS] and Mental Component Score [MCS] are calculated.

#### Fear-Avoidance Beliefs Questionnaire (FABQ)

This is a 16 item (0-6 scale per item) self-report questionnaire that focuses on participants beliefs about how physical activity (5 items; 0-30) and work (11 items; 0-66) affect their low back pain [71].

#### Hospital Anxiety and Depression Scale (HAD)

This is a widely used self-report questionnaire for detecting overall states of anxiety and depression in non-psychiatric medical contexts [72]. It consists of 14 items which are statements to be scored on 4-point Likert-scales (0-3), generating 'Anxiety' or 'Depression' scores ranging from 0 to 21 (total score = 0-42); which are categorised as a "non-case" (0-7 points), a "borderline case" (8-10 points) or a "case" (≥11 points).

#### International Physical Activity Questionnaire (IPAQ)

This is a self or telephone-administered physical activity recall questionnaire, which will ask participants about the time they spent being physically active in the previous seven days. It has been found to be a valid method for monitoring population levels of physical activity globally for populations of 18-69 years of age [73].

#### Exercise Self Efficacy Questionnaire

Participants will be requested to rate their confidence in exercising under five different situations (i.e. when tired, in a bad mood, limited time, on holiday, bad weather) on five point Likert scales ranging from 'not at all confident' to 'extremely confident' [74].

#### Readiness to Change Questionnaire

Participants will be requested to state their current level of physical activity participation from one of five possible responses ranging from 'I currently do not exercise and I do not intend to start exercising in the next 6 months' to 'I currently exercise regularly and have done so for longer than 6 months' [75].

#### Employment status

The employment status of all participants (employed, homemaker, carer, unemployed, student, retired, disability) and current work status (working, sick leave) of those in paid employment only will be recorded at each follow-up point.

#### Patient Satisfaction Questionnaire

This questionnaire utilises Likert scales to assess participant satisfaction with outcome and satisfaction with physiotherapy care during the trial and will be administered at the 3 month follow-up point only [76]

#### Devices Utility Questionnaire

This is 7-item questionnaire designed to establish the utility (user-friendliness and its difficulties) of the objective sleep measures, adapted from a previously developed questionnaire [77]. Acceptability as a sleep measure, interference with sleep, wearing comfort, and awareness of wearing the device will be scored on 11-point numerical rating scales from 0 (not acceptable) to 10 (very acceptable). Participants will be requested to provide any free text comments on the use of the objective measures.

Participants will be phoned to request attendance at a face to face follow-up in the physiotherapy department at 3 months, and pre-paid postage envelopes will be sent at 6 months. Follow-up reminders will be given by phone and letter. Non-responders to the 3 month face to face follow-up will be requested to return completed questionnaires by pre-paid post or by telephone. A courier will be available to return the sleep monitoring devices unreturned by patients during the trial.

### Interventions

#### (i) Supervised exercise class (SEC)

Within one week of randomisation, participants will commence the SEC. This class will follow a group-based format based on the 'Back to Fitness' programme used in the UK BEAM trial [37] which is underpinned by cognitive behavioural therapy principles designed to change participants behaviour by modifying their attitude to their LBP, i.e. 'hurt' does not mean harm [78,79]. First, each participant will attend the physiotherapy department for an initial individual assessment with the Chartered Physiotherapist delivering the class, where there will be discussion and agreement between the therapist and the patient on short and long-term goals; recording of the patient's exercise capabilities and perceived barriers to recovery and the individual's treatment expectations. Second, participants will attend the physiotherapy department of the relevant participating hospital once a week for 8 weeks for a one-hour supervised group exercise class led by a Chartered Physiotherapist. The physiotherapist will advise patients according to their individual goals and exercise capabilities, and help identify which exercise(s) they could continue independently of the treatment sessions, i.e. foster the development of self-management strategies. Participants will also be required to rate their perceived exertion during the class on the Borg scale - a linear scale measuring level of breathlessness from 0 = 'not breathless at all' to 10 = 'maximal' [80,81]. Patients will be encouraged to accept responsibility for determining and carrying out their weekly programme of activity. Adherence with the supervised exercise programme will be recorded as the number of sessions attended. The number of sessions defined as adherence will be decided on completion of the trial.

#### (ii) Walking programme (WP)

Within one week of randomisation, participants will commence the WP, the focus being to increase physical activity through a graded walking programme. The WP is based on previous effective programmes in healthy sedentary adults [82-85] and its clinical and cost effectiveness is currently being evaluated in a multi-centre randomized controlled trial involving people with chronic LBP [39].

As with the SEC, each participant will attend the physiotherapy department for an initial individual assessment, where there will be discussion and agreement between the therapist and the patient on short and long-term goals; recording of the patient's exercise capabilities and perceived barriers to recovery and the individual's treatment expectations. Participants allocated to the WP will be given an educational walking manual and requested to record habitual daily activity levels (frequency of walks, walk duration) an exercise diary prior to the start of the intervention. Participants will be given an educational walking manual, and a Yamax Digiwalker Pedometer Model SW-200 and following instruction in its use requested to record the number of steps in an exercise diary. The starting point for the eight week progressive WP will be a minimum of a 10 minute walk (approx 1200 steps) on at least four days per week to be decided with, where possible, one day's rest between walks, on the basis of each participant's normal physical activity levels during the first week of recording.

The aim of the programme is to progress to the American College of Sports Medicine guidelines of 30 minutes moderate intensity walking on five days per week by week five [86], and then to maintain this level for the remainder of the programme. The 30 minutes brisk walking may be accumulated in two or three shorter bouts if this is more attainable e.g. three 10 minute walks [83,85]. A recent review found no difference in the positive effects on cardiovascular fitness of empirical studies of accumulated or continuous physical activity in sedentary adults and highlighted the need for research to evaluate if accumulated exercise may increase compliance in previously sedentary adults [87]. All participants will be encouraged to use the Borg Breathlessness scale to establish their walking speed: targeting level three (moderate breathlessness) to four (somewhat severe), the minimum level required to achieve the benefits related to exercise [80,81].

Participants will then be contacted once per week by telephone by the Chartered Physiotherapist who performed the initial assessment to progress their walking frequency and duration based on their exercise diary record of the previous week's walking, and to provide encouragement. These telephone calls will be based around a telephone script based upon CBT principles, adapted from a previously developed telephone script for another LBP clinical trial [88]. Participants will be advised that just like athletes, any unaccustomed exercise is likely to produce some muscle soreness [79]. Participants will be advised to use their pedometer [89] as a motivational feedback tool, providing immediate information on activity levels [90].

Adherence with the walking programme will be assessed by the frequency, distance, number of steps taken and duration of walks recorded in the exercise diary. Specific adherence levels will be established once the trial is complete. At the end of the intervention participants will re-attend the physiotherapy department for a review appointment with a view to discharge from physiotherapy.

#### (iii) Usual Physiotherapy (UP) -Control Group

Within one week of randomization, participants will commence individual usual physiotherapy at the discretion of the treating physiotherapist. All physiotherapy treatments and the number of visits will be recorded for the study period in previously designed treatment record forms. On the basis of a previous RCT by the Principal Investigator in the Republic of Ireland public physiotherapy health service the anticipated mean (SD) number of treatments is 5.8 over a mean (SD) of 7.7 weeks (5.8) weeks [91]. A multimodal approach of education/advice, manipulative therapy and exercise therapy will be permitted on the basis of the results of previous surveys of physiotherapy practice in the UK and Ireland [25-28]. As part of this it is expected that participants will be provided with an individualized exercise programme at the discretion of the treating therapist but will not be permitted to attend group exercise classes or undertake a walking programme during the trial. Adherence will be assessed by the number of visits prior to discharge from physiotherapy.

### Adverse effects or events

No adverse events, apart from minor musculoskeletal complaints in the WP group, are anticipated but will be documented by type, length of time, and frequency should they occur [92].

### Data Analysis

All data will be coded and entered into the Statistical Package for the Social Sciences database for analysis following data cleaning and checking for errors. Since this is a feasibility study extensive exploratory analysis of the data will be undertaken. Treatment effects will be represented by point estimates and confidence intervals of all outcome variables at all follow-up points. Means and standard deviations will be used to calculate effect sizes for the main outcome variables (i.e. PSQI, ODI), and to undertake a power calculation for the main trial. Monthly recruitment rates and ratio of number screened: number enrolled will be tabulated. This information will be used to help select the recruitment period and number of centres for the main study. The assessment of patient satisfaction will be tabulated, as will adherence levels, any recorded difficulties experienced with the protocol, including the use of the sleep monitoring equipment or adverse events experienced by the patients or therapists. The following criteria would suggest that a main trial is not feasible: no apparent change in the outcomes with confidence intervals that include large negative values, feedback from participants that they were unable/unwilling to complete the outcome measures or adhere to the intervention, high level of adverse events.

### Qualitative study

All participants will receive an invitation letter to participate in a semistructured telephone interview at the end of the 6 month follow-up. The telephone interviews will be conducted by an experienced interviewer with a pre-determined set of questions. A "clue and process" format using a checklist of topics, will be used to ensure that the same basic areas are covered but allowing any issues of importance to the participants to emerge. The sessions will be audiotaped, minuted and transcribed verbatim for independent analysis of emergent themes. The main areas to be explored will be the effect of back pain on participant's sleep, reasons for participation in the trial, their interpretation of study information and documentation, their views on the methods of sleep monitoring used in the trial, their experiences, expectations and satisfaction with the programme of care including barriers/motivators to participation in the relevant programme and the impact of the intervention on sleep.

### Data Analysis

Qualitiative data from the telephone interviews will be analysed using Burnard's thematic analysis [93]. This process aims to produce a systematic and detailed recording of the themes addressed in the interviews and to link the themes and interviews together under a reasonably exhaustive category system. Emerging themes will be identified and comparisons explored between participants' experience of trial participation including monitoring of their sleep, their perception of treatment effectiveness and response to each intervention, its impact, motivators and barriers to adherence, as well as their expectations and treatment preferences. A random sample of transcripts from each group will be selected and reviewed by an independent researcher not otherwise involved in the study for inter-rater and intra-rater reliability of identified themes (trustworthiness, internal validity).

## Discussion

We have presented the rationale and design of a feasibility randomised controlled trial to evaluate the effectiveness of three forms of physiotherapy (a supervised general exercise programme, a walking programme, and usual physiotherapy) for sleep disturbance in participants with chronic low back pain. The results of this study will be presented as soon as they are available.

## Abbreviations

The following abbreviations have been used in the manuscript: LBP: low back pain; CLBP: chronic low back pain; RCT: randomized controlled trial; CBT: cognitive behavioural therapy; ODI: Oswestry Disability Index; PSQI: Pittsburgh Sleep Quality Index; UK: United Kingdom; SEC: supervised exercise class; WP: walking programme.

## Competing interests

The authors declare that they have no competing interests.

## Authors' contributions

DAH conceived the study. All authors were involved in aspects of the design of the study. DAH will act as Principal Investigator and was responsible for drafting the paper, and all authors commented on the draft. All authors have read and approved the final manuscript.

## Pre-publication history

The pre-publication history for this paper can be accessed here:

http://www.biomedcentral.com/1471-2474/11/70/prepub
